# Enhanced malic acid production from glycerol with high-cell density *Ustilago trichophora* TZ1 cultivations

**DOI:** 10.1186/s13068-016-0553-7

**Published:** 2016-07-02

**Authors:** Thiemo Zambanini, Wiebke Kleineberg, Eda Sarikaya, Joerg M. Buescher, Guido Meurer, Nick Wierckx, Lars M. Blank

**Affiliations:** Institute of Applied Microbiology – iAMB, Aachen Biology and Biotechnology – ABBt, RWTH Aachen University, Worringerweg 1, Aachen, 52074 Germany; BRAIN AG, Zwingenberg, 64673 Germany

**Keywords:** Bioreactor, Fed-batch, Crude glycerol, Malic acid, *Ustilago trichophora*

## Abstract

**Background:**

In order to establish a cost-efficient biodiesel biorefinery, valorization of its main by-product, crude glycerol, is imperative. Recently, *Ustilago trichophora* TZ1 was found to efficiently produce malic acid from glycerol. By adaptive laboratory evolution and medium optimization, titer and rate could be improved significantly.

**Results:**

Here we report on the investigation of this strain in fed-batch bioreactors. With pH controlled at 6.5 (automatic NaOH addition), a titer of 142 ± 1 g L^−1^ produced at an overall rate of 0.54 ± 0.00 g L^−1^ h^−1^ was reached by optimizing the initial concentrations of ammonium and glycerol. Combining the potential of bioreactors and CaCO_3_ as buffer system, we were able to increase the overall production rate to 0.74 ± 0.06 g L^−1^ h^−1^ with a maximum production rate of 1.94 ± 0.32 g L^−1^ reaching a titer of 195 ± 15 g L^−1^. The initial purification strategy resulted in 90 % pure calcium malate as solid component. Notably, the fermentation is not influenced by an increased temperature of up to 37 °C, which reduces the energy required for cooling. However, direct acid production is not favored as at a lowered pH value of pH 4.5 the malic acid titer decreased to only 9 ± 1 g L^−1^. When using crude glycerol as substrate, only the product to substrate yield is decreased. The results are discussed in the context of valorizing glycerol with Ustilaginaceae.

**Conclusions:**

Combining these results reveals the potential of *U.trichophora* TZ1 to become an industrially applicable production host for malic acid from biodiesel-derived glycerol, thus making the overall biodiesel production process economically and ecologically more feasible.

## Background

The production of biodiesel, as one possible supplement for petroleum-derived fuels, is a great opportunity to drive the needed switch to a bio-based economy. This is also reflected in the ever increasing amount of produced biodiesel, which is predicted to be 123 million tons per year for 2016 [1]. However, this process results in a 10 % (w/v) waste stream of crude glycerol, decreasing the profit margin and the ecological feasibility. Valorization of this large low-value side stream by microbial conversion is considered as a promising strategy to add value to the overall biodiesel biorefinery concept. Microbial production processes starting from glycerol as substrate have been investigated and reviewed intensively over the last years resulting in production processes for many different products [2–4].

The C_4_-dicarboxylic acid malic acid is widely used as acidulant and flavor enhancer in the food industry and has also received great interest in non-food applications, such as metal cleaning, textile finishing, and pharmaceuticals production [5]. Even though the annual world production in 2006 was only about 40,000 tons, future use of malic acid is predicted to be above 200,000 tons per year as raw material of a novel biodegradable polymer–polymalic acid [5, 6]. In 2004, malic acid has been identified by the Department of Energy (DOE) as one of the top twelve building block chemicals to be produced from renewable biomass at bulk scale [7]. Traditionally, malic acid was obtained by the extraction from apple juice at low yields [8]. Today malic acid can be produced both chemically and biotechnologically. In current industrial production processes, it is mainly manufactured by chemical synthesis via hydration of maleic or fumaric acid producing a racemic mixture of d- and l-isomers [9]. Alternatively, enzymatic hydration of fumarate by immobilized bacterial cells of *Brevibacteriumammoniagenes* or *Bacillusflavum* containing a highly active fumarase yields enantiomerically pure l-malic acid [10]. However, these production methods are costly and substrates for the synthesis of malic acid are derived from non-sustainable petrochemical feedstocks [5]. Thus, as TCA cycle intermediate, bio-based microbiological production processes based on renewable substrates for malic acid have become the focus of research. The first patented microorganism producing malic acid was *Aspergillusflavus* [11]. The fermentation process was improved by medium optimization resulting in a final titer of 113 from 120 g L^−1^ glucose as substrate [8]. However, this organism is not applicable for industrial malic acid production, especially for food applications, due to the production of aflatoxins [12]. Besides *Escherichiacoli* [13, 14] and *Saccharomycescerevisiae* [15], an *Aspergillusoryzae* strain has been investigated as production organism. This strain, overexpressing a C_4_-dicarboxylate transporter, pyruvate carboxylase, and malate dehydrogenase produced a final titer of 154 g L^−1^ malic acid from glucose at a rate of 0.94 g L^−1^ h^−1^ [16].

Recently we reported that *Ustilagotrichophora* TZ1, a member of the family of Ustilaginaceae which is known to produce organic acids naturally [17], is able to produce malic acid from glycerol [18]. This strain has been adapted to glycerol by laboratory evolution, increasing glycerol uptake rates. After medium optimization, the final malic acid titer reached 196 g L^−1^ produced from 250 g L^−1^ glycerol at an average rate of 0.4 g L^−1^ h^−1^ in shake flasks. The limiting factor in these shake flask cultivations was either glycerol depletion or problems concerning oxygen transfer, which result from viscous culture broth.

Here we report on malic acid production with *U.trichophora* TZ1 in bioreactors to overcome abovementioned problems. Further, the production process was investigated at differing temperature profiles and pH values to determine the boundary conditions of an eventual industrial process, and the effects of using high concentrations of crude glycerol as a substrate were evaluated.

## Results and discussion

### Bioreactors enable higher cell density resulting in higher volumetric production rates

The potential of Ustilaginaceae as production organisms of different industrially relevant compounds, such as organic acids, lipids, or polyols, has been discussed and demonstrated consistently over the last years [17, 19–25]. Recently, *U.trichophora* was found to produce malic acid naturally from glycerol at high titers. By adaptive laboratory evolution and medium optimization, the production rate of this strain in shake flask could be improved to around 0.4 g L^−1^ h^−1^ reaching titers near 200 g L^−1^ [18]. All cultivations ended either upon glycerol depletion or by oxygen limitations due to the viscosity of the cultures. These viscosity issues resulted mainly from the buffering agent, CaCO_3_, reacting with produced malate, forming insoluble calcium malate. Although this precipitation might be beneficial for alleviation of product inhibition, it greatly hinders oxygenation of the culture broth in shaking flasks [26].

To overcome handling issues with insoluble components and to avoid glycerol depletion, here we investigate the production process with *U.trichophora* TZ1 in bioreactors, in which the pH was kept constant by titration with NaOH. By this, effects of insoluble buffer components on production can be minimized. Further, by feeding additional glycerol prior to depletion, malate titers might be further increased. Additionally, better oxygenation through sparging and stirring, which has a strong influence on microbial organic acid production processes [27], also enables higher cell densities.

**Fig. 1:**
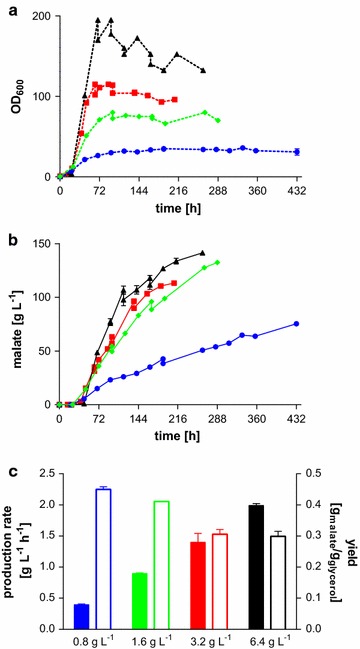
Fermentation of *Ustilago* *trichophora* TZ1 with different NH_4_Cl concentrations. **a** OD_600_, **b** malate production, **c** maximum malate production rate (*solid bars*) and yield (*open bars*) for controlled batch fermentations in MTM containing 200 g L^−1^ initial glycerol at 30 °C and pH 6.5 with DO kept at 80 %. *Colors* indicate different initial NH_4_Cl concentrations: 0.8 g L^−1^ (*circles*, *blue*), 1.6 g L^−1^ (*diamonds*, *green*), 3.2 g L^−1^ (*squares*, *red*), and 6.4 g L^−1^ with doubled concentrations of all medium components except glycerol (*triangles*, *black*). Values for 0.8 g L^−1^ are only shown until 432 h; however, a further increase in concentration to a final titer of 120 ± 1 g L^−1^ could be observed until 908 h of cultivation. *Error bars* indicate deviation from the mean (*n* = 2)

Initially, *U.trichophora* TZ1 was cultured in pH controlled bioreactors (pH 6.5, NaOH titration) in MTM containing 0.8 g L^−1^ NH_4_Cl and 200 g L^−1^ initial glycerol. An additional 160 g glycerol was fed when the concentration dropped below 50 g L^−1^. This results in a slight drop in the measured malate concentrations due to the dilution of the culture broth. The resulting titer (119.9 ± 0.9 g L^−1^) and rate (0.13 ± 0.00 g L^−1^ h^−1^) (Fig. 1b) were significantly lower than those reached in shake flasks with CaCO_3_ [18]. Likely, these reductions can be attributed to product inhibition caused by the drastically increased dissolved malate concentration in NaOH-titrated cultures. To improve the production rate, the cell density was increased by using higher concentrations of the growth-limiting nutrient NH_4_Cl (1.6, 3.2, and 6.4 g L^−1^). Dependent on the initial NH_4_Cl concentration, a delay in the onset of malate production could be observed, which can be attributed to a longer growth phase. Maximal OD_600_, however, could be increased from 42 ± 2 with 0.8 g L^−1^ NH_4_Cl to 80 ± 0 and 115 ± 1 using 1.6 and 3.2 g L^−1^ NH_4_Cl, respectively (Fig. 1a). As expected, also the overall volumetric malic acid production rate (from the beginning of cultivation until the end) increased to 0.46 ± 0.02 and 0.54 ± 0.07 g L^−1^ h^−1^ with 1.6 and 3.2 g L^−1^ NH_4_Cl, respectively (Fig. 1b). 6.4 g L^−1^ NH_4_Cl, however, did not lead to increased biomass and subsequently production, but had the opposite effect (data not shown). In these cultures, NH_4_Cl was no longer depleted during the fermentation. A similar effect was observed for itaconate producing *Ustilago maydis* MB215 in MTM with NH_4_Cl concentrations above 4 g L^−1^ [19]. This likely explains the reduced productivity, since nitrogen limitation is the most efficient trigger for organic acid production with Ustilaginaceae [28]. To compensate for this effect, all medium components except for glycerol were doubled in combination with 6.4 g L^−1^ NH_4_Cl in a subsequent fermentation, resulting in an overall volumetric production rate of 0.54 ± 0.00 g L^−1^ h^−1^, with a maximal production rate of 1.99 ± 0.04 g L^−1^ h^−1^ between 45 and 69 h (Fig. 1b).

As expected, an increase in the growth-limiting nutrient led to more biomass formation and consequently to a higher volumetric production rate. There is a good correlation between the maximum malate production rate and the initial NH_4_Cl concentration, indicating that the production rate could be further increased as long as secondary limitations are excluded. However, further increases will strongly impact the product yield, since more glycerol is used for biomass formation. Assuming no CO_2_ co-consumption, the maximum theoretical yield would be 0.75 mol malate per mole glycerol. However, the glycerol needed for biomass production reduces this maximum, and this reduction is proportional to the initial ammonium concentration. Based on the glycerol consumption during the growth phase (Fig. 1a), approximately 11.5 g of glycerol are needed for biomass formation per gram NH_4_Cl. Thus, taking into account the total amount of glycerol consumed by each culture, biomass formation reduces the maximum theoretical yield to 0.73, 0.71, 0.68, and 0.62 mol mol^−1^, for 0.8, 1.6, 3.2, and 6.4 g L^−1^ NH_4_Cl, respectively. This in part explains the reduction in the observed yields in the cultures with higher NH_4_Cl concentrations, although in general the yields are only 30–55 % of these theoretical maxima, suggesting that the impact of biomass formation is at the moment relatively low. Improvement in the product yield should be the main focus of future optimization, possibly by the reduction in by-product formation through the disruption of competing pathways. The improvement in specificity for the production of one organic acid is generally considered a promising approach to improve microbial organic acid production. For *U.trichophora* TZ1, however, besides 5–10 g L^−1^ succinate, no significant amounts of other organic acids were found in HPLC analysis. Additionally, CO_2_ and extra- and intracellular lipids are most likely the main by-products. The formation of lipids under organic acid production conditions and their effect on the cells have been described extensively [28, 29]. These by-products can be reduced by knock-out of single genes in the responsive gene clusters [30–32].

**Fig. 2:**
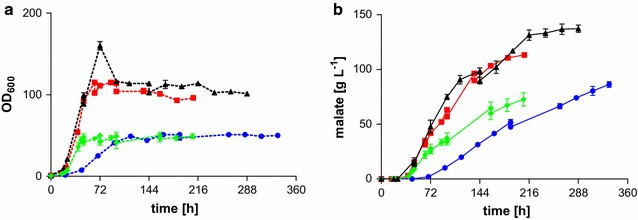
Fermentation of *Ustilago* *trichophora* TZ1 with different initial glycerol concentrations. **a** OD_600_, **b** malate production for fermentations in MTM containing 3.2 g L^−1^ NH_4_Cl at 30 °C and pH 6.5 with DO kept at 80 %. *Colors* indicate different initial glycerol concentrations: 300 g L^−1^ (*circles*, *blue*), 250 g L^−1^ (*diamonds*, *green*), 200 g L^−1^ (*squares*, *red*), 150 g L^−1^ (*triangles*, *black*). Additional 160 g glycerol was added when the concentration dropped below 50 g L^−1^. *Error bars* indicate deviation from the mean (*n* = 2)

Since a significant influence of the starting glycerol concentration on the malic acid production rate has been observed in shake flasks [18], this relation was also studied in bioreactors. Concentration steps of 50 g L^−1^ between 150 and 300 g L^−1^ were investigated in MTM containing 3.2 g L^−1^ NH_4_Cl. Additional 160 g glycerol was fed to the cultures one time (300 g L^−1^ initial glycerol), two times (150 and 200 g L^−1^ initial glycerol), and four times (250 g L^−1^ initial glycerol), when the concentration became lower than 50–100 g L^−1^ (150 and 200 g L^−1^ initial glycerol) or 200 g L^−1^ (250 and 300 g L^−1^ initial glycerol). Thus, after the consumption of the initial glycerol, its concentrations generally ranged between 50 and 150 g L^−1^ (150 and 200 g L^−1^ initial glycerol) and 100 and 250 g L^−1^ (250 and 300 g L^−1^ initial glycerol). Just as in shake flasks, increasing initial glycerol concentrations between 150 and 300 g L^−1^ decreased growth rates, final OD_600_ and malic acid production rates (Fig. 2). Possibly, higher glycerol concentrations impose a stress upon the cells. This is also known in other organisms, such as *S.cerevisiae*, even though lower glycerol concentrations are generally known to contribute to osmotolerance in different yeast, such as *Zygosaccharomycesrouxii* and *S.cerevisiae* [33, 34].

### *Ustilago trichophora* TZ1 accepts a broad temperature range for production

**Fig. 3:**
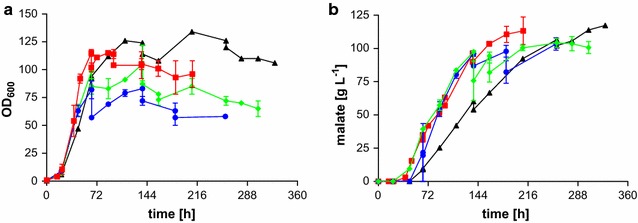
Fermentation of *Ustilago* *trichophora* TZ1 at different temperatures. **a** OD_600_, **b** malate concentration for fermentations in MTM containing 200 g L^−1^ initial glycerol and 3.2 g L^−1^ NH_4_Cl at 30 °C and pH 6.5 with DO kept at 80 %. *Colors* indicate different temperatures: 25 °C (*triangles*, *black*), 30 °C (*squares*, *red*), 35 °C (*circles*, *blue*), and 37 °C (*diamonds*, *green*). *Error* *bars* indicate deviation from the mean (*n* = 2)

In 1990, Guevarra and Tabuchi investigated the influence of temperature on itaconic acid production and growth of *Ustilagocynodontis* [35]. They could show that the highest tested temperature (35 °C) was best for cell growth. However, the lowest tested temperature (25 °C) resulted in the highest organic acid titers. To investigate influences of temperature on acid production by *U.trichophora* TZ1, cells were grown at 30 °C and the temperature was changed after the growth phase to 25 and 35 °C. In a third approach, heating was disabled and cooling was only activated at temperatures exceeding 37 °C (Fig. 3). In this case, the temperature remained at this maximum after 30 h, indicating the considerable heat generated by these high-density cultures. As shown in Fig. 3b, malic acid production was not influenced by temperatures exceeding 30 °C. However, 25 °C resulted in a lower malic acid production rate yet reaching the same final titer of approximately 120 g L^−1^.

Since malic acid production with *U.trichophora* TZ1 was not influenced by elevated temperatures and reduced use of heating and cooling systems could reduce operating costs, preliminary experiments without a heating and cooling system were conducted. These experiments indicated that uncontrolled temperatures above 37 °C negatively influence the malic acid production process. This was also observed in 2008 by Kuenz for itaconic acid production with *Aspergillusterreus* [36]. A temperature increase from 27 to 30 °C resulted in a 60 % increased production rate. Further increasing the temperature to 33 and 37 °C resulted in a 20–40 % increase compared to 30 °C. However, a process temperature of 40 °C reduced itaconic acid production drastically [36].

### Decreasing pH values drastically lower malic acid production

**Fig. 4:**
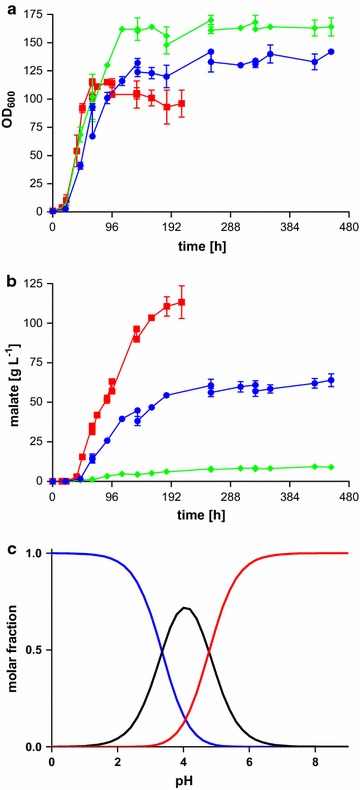
Fermentation of *Ustilago* *trichophora* TZ1 at different pH values. **a** OD_600_, **b** malate concentration for fermentations in MTM containing 200 g L^−1^ initial glycerol and 3.2 g L^−1^ NH_4_Cl at 30 °C and pH 6.5 with DO kept at 80 %. Additional 160 g glycerol was added when the concentration dropped below 50 g L^−1^. *Colors* indicate different pH values: pH 6.5 (*red*), 5.5 (*blue*), and 4.5 (*green*). *Error* *bars* indicate deviation from the mean (*n* = 2). **c** Distribution of molar fractions of dissociated and (partly) undissociated malate species. Shown is the relative distribution of fully dissociated (*blue*), partially dissociated (*black*) and fully undissociated (*red*) malate dependent on the pH value. Data were generated using CurTiPot [56]

In a next step, the fermentation was investigated in respect to growth medium pH. Malic acid production with *U.trichophora* TZ1 was investigated in bioreactors at pH 4.5, 5.5, and 6.5. The tested pH range neither influenced growth rate (Fig. 4a), nor morphology (data not shown). However, maximal OD_600_ was higher at lower pH. Malic acid production was clearly lowered by decreasing pH reaching 113 ± 15 g L^−1^ (pH 6.5), 64 ± 6 g L^−1^ (pH 5.5), and 9 ± 1 g L^−1^ (pH 4.5). In fungi such as *Aspergillus*, *Saccharomyces*, and *Yarrowia*, organic acids such as succinate, itaconate, and malate are produced best at low pH, with some exceptions [27, 37–41]. For Ustilaginaceae, mainly near neutral pH values are best for organic acid production [19], although exceptions such as *U.cynodontis* have been reported [17].

Production both at high and at low pH value has different opportunities and disadvantages on microbial organic acid production and downstream processing. A low pH can help to lower the risk of contamination in industrial-scale fermentations. Further, the production of environmentally unfriendly by-products can be reduced, since during the production process less titration agents, such as CaCO_3_ or Ca(OH)_2_, are needed, which in the later process have to be disposed of. However, the same by-product, namely gypsum, is also produced in the downstream process of microbial citric acid production, resulting from the reaction of sulfuric acid with calcium–citrate [42]. However, more advanced downstream technologies, such as simulated moving bed [43], are becoming ever more established and could enable a calcium-free process, provided that it does not negatively impact the overall process efficiency. Another advantage of producing acids at low pH is the easier downstream processing itself, since methods such as cooling, evaporation–crystallization or salting [20, 44] are possible. Besides the positive effects of production at a low pH, there are many advantages for production at near neutral pH. One of those beneficial effects for Ustilaginaceae is the lowered burden, normally resulting from undissociated acids or low pH itself. Other advantages are the avoidance of thermodynamic constraints on acid export or the possibility of advanced process strategies, such as simultaneous saccharification and fermentation (SSF) in which the pH optimum of the applied enzymes is essential [6, 28, 45].

pH values near the lower *p*Ka value of malate (*p*Ka_1_ 3.46, *p*Ka_2_ 5.10) [15] result in undissociated malic acid. Although the molar fraction of this undissociated species is relatively low (approximately 0.002 % at pH 6.5, 0.1 % at pH 5.5 and 4.8 % at pH 4.5; Fig. 4c), its protonophoric effect likely disrupts cellular pH homeostasis. This, possibly coupled to an increased intracellular malic acid concentration, likely leads to the observed reduction in malate production. The weak acid uncoupling effect caused by uptake of the protonated form via diffusion with a simultaneous import of a proton and needed active transport of the dissociated form out of the cell leads to a loss of energy [45, 46]. A further loss of energy can result from the export mechanism itself. It was reported that the most likely mechanism for export of dicarboxylic acids at low pH is an antiport with protons [47]. This would lead to additional H^+^ ions pumped against the proton motive force, which consequently increases ATP consumption [48]. The observation that glycerol uptake is not decreased in cultures with lower pH, would strengthen this hypothesis, since its consumption could help to cope with the energy loss.

### CaCO_3_ as buffering agent helps to overcome product inhibition

Independent from final OD_600_, malic acid production, glycerol consumption, growth rate, and temperature, a clear drop in production rate at malate concentrations above 100 g L^−1^ is visible and the maximal titer of around 140 g L^−1^ was not exceeded. In shake flask cultivations containing CaCO_3_ as buffer agent, however, this titer had been exceeded with constant production rates until glycerol depletion [18]. In these cultures, the CaCO_3_ reacts with the produced malic acid forming calcium malate, which precipitates at a concentration above 14 g L^−1^. As a consequence, additionally produced malate is no longer dissolved in the medium, thus alleviating product inhibition and toxicity. These results strongly suggest a negative effect of product inhibition at concentrations above 100 g L^−1^.

**Fig. 5:**
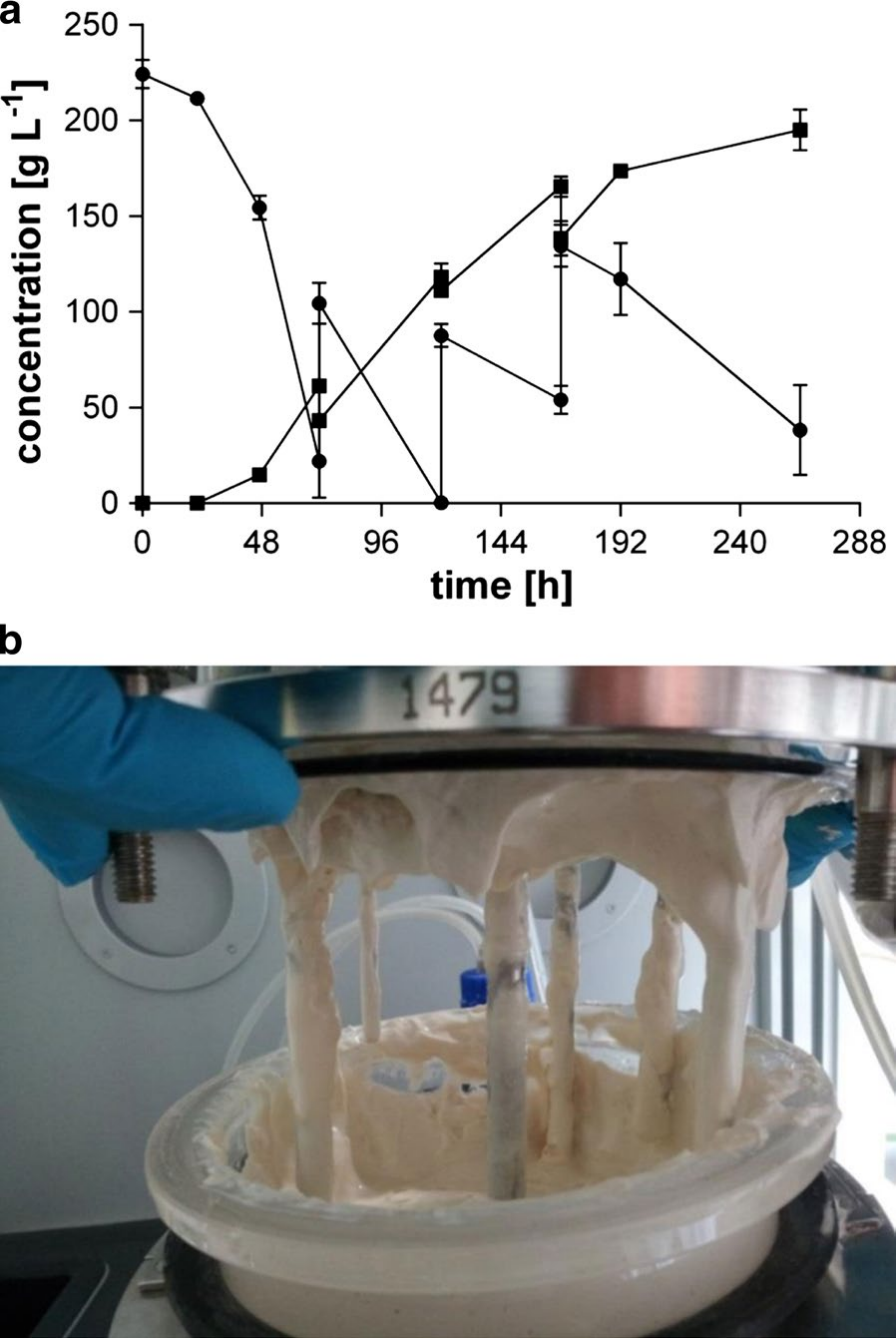
Fermentation of *Ustilago* *trichophora* TZ1 with CaCO_3_. **a** malate concentration (*squares*) and glycerol concentration (*circles*), **b** fermentation broth after 264 h of fermentation in MTM containing 200 g L^−1^ glycerol, 3.2 g L^−1^ NH_4_Cl and 100 g L^−1^ initial CaCO_3_ at 30 °C with DO kept at 80 %

To overcome the assumed product inhibition in fed-batch bioreactors, cultivations with MTM containing 3.2 g L^−1^ NH_4_Cl, 200 g L^−1^ initial glycerol and 100 g L^−1^ CaCO_3_ as buffer were performed (Fig. 5). An additional 150 g L^−1^ CaCO_3_ was added when the pH dropped below 5.5 and additional 160 g glycerol was fed when the concentration fell below 50 g L^−1^. This fermentation resulted in the production of 195 ± 15 g L^−1^ of malic acid within 264 h of cultivation, corresponding to an overall production rate of 0.74 ± 0.06 g L^−1^ h^−1^. The process reached a yield of 0.43 ± 0.05 g_mal_ g_gly_^−1^ and a maximal production rate of 1.94 ± 0.32 g L^−1^ between 47 and 71 h (Fig. 5a). Both glycerol consumption and malic acid production decreased over time. The yield during production phase, however, stayed constant in a range of 0.39–0.49 g_mal_ g_gly_^−1^, indicating that the decreasing production rate is rather an effect of dilution due to glycerol feed than an actual decrease in the specific productivity.

The yield achieved with CaCO_3_ as buffer is 1.5-fold higher than with NaOH. This increase may either be due to an increase in CO_2_ co-fixation through the action of pyruvate carboxylase or to a reduction in product inhibition by in situ crystallization of calcium malate. Based on the current yield, and assuming that all remaining glycerol is converted to CO_2_, 85 % of the total produced CO_2_ originates from glycerol. The remaining 15 % originates from CaCO_3_ (12 %) and aeration (3 %). Given this relatively low contribution of CaCO_3_ to the overall CO_2_ balance, a positive effect of additional CO_2_ co-metabolism from CaCO_3_ is unlikely. This suggests that the higher yield observed with CaCO_3_ is mainly due to reduction in product inhibition.

At 264 h, the fermentation had to be stopped due to bad mixing caused by high medium viscosity (Fig. 5b) as had already been experienced for shake flask cultivations using CaCO_3_ as buffering agent [18]. This increased viscosity, likely caused by calcium malate, results in poor and inhomogeneous oxygenation. Further, even though the formed calcium malate can easily be recovered for downstream processing, it is linked to a large stream of gypsum waste, which results from the reaction with sulfuric acid within the downstream process as already mentioned above [42]. This gypsum needs to be disposed of as environmentally unfriendly leftover of this process. However, the prior limit of 140 g L^−1^ malic acid in bioreactors could be exceeded, further sustaining the hypothesis of product inhibition at concentrations above 140 g L^−1^. Additionally, the malic acid production rate could be kept near constant for a longer time. These advantages have to be weighed against the abovementioned drawbacks in order to determine the beneficial effect of CaCO_3_ as buffering agent.

As already mentioned, the formation of solid calcium malate in bioreactors containing CaCO_3_ as buffering agent enables efficient initial purification. To isolate the product from the fermentations, all solid components (settled for 48 h) resulting from an autoclaved fermentation with CaCO_3_ (Fig. 5b) were dried at 120 °C for 24 h. 0.2 g of this mixture was dissolved in 1 mL of HCl (37 %) and adjusted to 2 mL with water in triplicates. The mixture was filtered to remove cells and the malate concentration was determined via HPLC to be 68.1 ± 0.1 g L^−1^. Assuming that all products are recovered in the form of calcium malate, this is nearly 90 % of the theoretical malic acid concentration (78 g L^−1^), indicating that the solids recovered from the bioreactor are 90 % pure calcium malate. The remaining 10 % can be assumed to be biomass and remaining CaCO_3_.

### *Ustilagotrichophora* TZ1 can cope with impurities in crude glycerol

Biodiesel-derived crude glycerol contains, depending on the biodiesel production process, impurities such as methanol, ash, soap, salts, non-glycerol organic matter, and water [2, 4]. Even though different microbial conversions of crude glycerol to value-added chemicals have been reported [49], many organisms struggle with the contained impurities, especially in fed-batch cultures with high substrate loads. The purification to pharma-grade glycerol, however, is a costly process often prohibiting the possible application of glycerol in microbial chemical production. To test whether *U.trichophora* TZ1 is able to cope with the contained impurities, we investigated malic acid production with *U.trichophora* TZ1 in MTM containing 100 and 200 g L^−1^ crude glycerol in shake flasks. The used crude glycerol contained 1.5 % ashes and 1.9 % free fatty acids, with a pH value between 6 and 8. Neither growth rate, nor maximal optical density, nor glycerol uptake was influenced by 100 and 200 g L^−1^ crude glycerol compared to the same amount of pharma-grade glycerol. Malic acid production, however, was lowered by 63 % (100 g L^−1^) and 41 % (200 g L^−1^) (data not shown). This indicates that the organism itself is capable of coping with the contained impurities, although at a cost resulting in a lower malic acid titer. This in shake flasks may be due to lower oxygen input as a result of increased salt concentrations, which can be up to 12 % in crude glycerol [4]. Increased osmotic pressure in media containing high concentrations of salts results in a lower maximum oxygen transfer rate in shake flasks [50]. The effect of this on growth and organic acid production was investigated in several organisms. For *U.maydis*, increased osmotic stress due to higher salt concentrations resulted in a prolonged lag-phase and lower growth rates. Interestingly, itaconic acid production slightly increased with higher salt concentrations [28], possibly due to high redox energy surplus generated with this product compared to malate. The same effect was observed in *Candidaoleophila* with increased citric acid production with higher osmolarity of the medium [51]. Since the redox potential of the different production pathways for malic acid, succinic acid and itaconic acid is completely different, the effect of reduced oxygen transfer rates will likely differ.

**Fig. 6:**
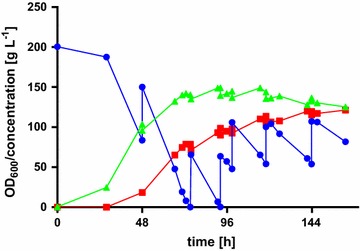
Fermentation of *Ustilago* *trichophora* TZ1 with crude glycerol. Malate concentration (*red*, *squares*), glycerol concentration (*blue*, *circles*) and OD_600_ (*green*, *triangles*) in MTM containing 200 g L^−1^ crude glycerol, 3.2 g L^−1^ NH_4_Cl at 28 °C (37 °C during production phase, 48 h) with DO kept at 30 %. pH was kept at 6.5 by automatic addition of NaOH. Shown is one exemplary fermentation run

To rule out this effect, we evaluated *U.trichophora* TZ1 in more industrially relevant conditions. To this end, it was cultivated in a bioreactor with MTM containing 200 g L^−1^ crude glycerol and 3.2 g L^−1^ NH_4_Cl. The pH was kept stable at 6.5 by automatic addition of NaOH. Additional crude glycerol was fed upon glycerol depletion (Fig. 6).

This fermentation resulted in OD_600_ values and growth rates comparable to the ones in bioreactors with pharma-grade glycerol. Also the glycerol uptake rate (2.90 g L^−1^ h^−1^) and the malic acid production rate (0.75 g L^−1^) were comparable to the ones with pharma-grade glycerol. Only the yield was lowered to 0.26 g g^−1^. A slight negative impact of crude glycerol compared to pharma-grade glycerol on organic acid production has already been shown for *Yarrowialipolytica* in citric acid production [52]. Interestingly, for *U.trichophora* TZ1 the accumulation of impurities by glycerol feed adding up to 476 g glycerol did not result in lowered production properties, which hints at an effect which is perhaps limited to the initial growth phase. A possibility to overcome this issue would be a second adaptive laboratory evolution on crude glycerol. For this, however, it has to be taken into consideration that depending on the origin of the crude glycerol, the composition of contained impurities differs in a broad range, not only in concentration, but also in components themselves [53]. In addition, to the already high tolerance to impurities in crude glycerol by *U.trichophora* TZ1 and thus only slight negative effect, the contained salts might also have a beneficial effect. For *Actinobacillussuccinogenes*, it could be shown that synthetic seawater can act as mineral supplement [54].

## Conclusions

The strain *U.trichophora* TZ1, which recently has been reported as promising production organism for malate from glycerol, is capable of producing 200 g L^−1^ malic acid at an overall rate of 0.74 g L^−1^ h^−1^ reaching a maximal production rate of 1.94 g L^−1^ h^−1^ and a yield of 0.31 mol mol^−1^ (31 % of the theoretical maximum assuming CO_2_ co-fixation or 41 % assuming no CO_2_ co-fixation) in bioreactors. These values, which are some of the highest reported for microbial malic acid production, allow *U.trichophora* TZ1, though only having undergone adaptive laboratory evolution and medium and fermentation optimization, to compete with highly engineered strains overexpressing major parts of the malate production pathway. Thus, further optimization of *U.trichophora* TZ1 could focus on metabolic engineering, which would not only harbor considerable potential to increase the production rate but also allow for strain optimization in terms of product to substrate yield by targeted disruption of by-product formation pathways. A subsequent systems biology comparison between the wild-type and the evolved strain not only could shed light on the adaptational mutations that enhanced the growth and production rate of *U.trichophora* TZ1 on glycerol but might also provide insights into why the strain utilizes glycerol faster than other Ustilaginaceae. In addition, it could clarify the glycerol uptake and degradation pathway and expand the general knowledge base of this relatively obscure *Ustilago* strain. This would clearly help to develop it into a platform for the production of not only malate but also other industrially relevant chemicals, to be produced from biodiesel-derived crude glycerol.

## Methods

### Strains and culture conditions

*Ustilagotrichophora* TZ1 was used throughout this study [18].

As standard medium, modified Tabuchi medium (MTM) according to Geiser et al. containing 0.2 g L^−1^ MgSO_4_ 7 H_2_O, 10 mg L^−1^ FeSO_4_ 7 H_2_O, 0.5 g L^−1^ KH_2_PO_4_, 1 mL L^−1^ vitamin solution, 1 mL L^−1^ trace element solution [17] and differing concentrations of NH_4_Cl and (crude) glycerol was used. For additional glycerol feeds, 200 mL of an 800 g L^−1^ glycerol solution was added to the cultures. Additional 150 g CaCO_3_ was fed to the cultures as solids, when the pH value dropped below 5.5. Pharma-grade glycerol was used for all cultures except for those where the use of crude glycerol is explicitly stated. Crude glycerol was used as 80 % (w/v) aqueous solution and autoclaved without prior purification. After addition of all medium components, the pH value was adjusted to 6.5.

All batch cultivations were performed in New Brunswick BioFlo^®^ 110 bioreactors (Eppendorf, Germany) with a total volume of 2.5 L and a working volume of 1.25 L. Temperature was maintained at 30 °C and the pH value was either set to 6.5 and controlled automatically with 10 M NaOH or different amounts of CaCO_3_ were added as buffer. To prevent foam formation, antifoam 204 (Sigma Life Science, USA) was added automatically using level sensor control. The aeration rate was set to 1.25 L min^−1^ (1 vvm) and the dissolved oxygen tension (DOT) was kept at 80 % saturation by automatically adjusting the stirring rate. As preculture, 50 mL MTM containing 0.8 g L^−1^ NH_4_Cl, 50 g L^−1^ glycerol, and 100 mM MES in 500-mL shake flasks was inoculated from an overnight YEP culture to an OD_600_ of 0.5. This culture was grown over night, washed twice by dissolving the pelleted cells (5000 rpm, 5 min, 30 °C) in 10 mL distilled water and used for inoculation of the bioreactor to an initial OD_600_ of 0.5. All shake flask cultures were incubated at 30 °C (relative air humidity = 80 %) shaking at 200 rpm (shaking diameter = 25 mm).

### Analytical methods

All experiments were performed in duplicates. Shown is the arithmetic mean of the duplicates. Error bars and ±values indicate deviation from the mean.

From bioreactors, 5 mL of culture broth was taken for OD_600_ and HPLC analysis. When using CaCO_3_ as buffer, the CaCO_3_ in 1 mL culture broth was dissolved with HCl prior to further measurements. OD_600_ was determined in an Ultrospec 10 cell density meter (Amersham Biosciences, UK); samples were diluted to an OD_600_ between 0.1 and 0.8.

For HPLC analysis, centrifuged samples (13.000*g*, 5 min) were filtered through cellulose acetate filters (diameter 0.2 µm, VWR, Germany) prior to diluting 1:10 with distilled water. For analysis of glycerol and organic acids, a Dionex Ultimate 3000 HPLC (Dionex, USA) was used with an Organic Acid Resin column (CS-Chromatographie, Germany) at 75 °C, with a constant flow rate of 0.8 mL min^−1^ 5 mM sulfuric acid as eluent. For detection, a Shodex RI 101 detector at 35 °C and a variable wavelength UV detector (Dionex, USA) at 210 nm were used.

Ammonium concentration was determined by a colorimetric assay according to Willis [55].

Calculation of the molar fraction of undissociated and dissociated species for malate was performed using CurTiPot [56].

## Abbreviations

MTM: Modified Tabuchi medium
MES: 2-(*N*-morpholino)ethanesulfonic acid
HPLC: High-performance liquid chromatography
